# Protocol for a systematic review to identify and weight the indicators of risk of asthma exacerbations in children aged 5–12 years

**DOI:** 10.1038/npjpcrm.2016.88

**Published:** 2017-01-05

**Authors:** Nara Tagiyeva, Susannah McLean, Aziz Sheikh, Steven Julious, Mike Thomas, James Paton, Hilary Pinnock, Members of the ARC group, Hilary Pinnock, Aziz Sheikh, Steven Julious, Mike Thomas, James Paton, Nara Tagiyeva, Susannah McLean, Audrey Buelo, Melissa Goodbourn, Andrew Bush, Steve Cunningham, Jonathan Grigg, John Henderson, Jurgen Schwarze, Michael D Shields, Gwyn Davies, Andrew Wilson

**Affiliations:** 1 Asthma UK Centre for Applied Research, Usher Institute of Population Health Sciences and Informatics, The University of Edinburgh, Edinburgh, UK; 2 Medical Statistics Group, School of Health and Related Research, University of Sheffield, Sheffield, UK; 3 Medicine, University of Southampton, Southampton, UK; 4 School of Medicine, College of Medical, Veterinary and Life Sciences, University of Glasgow, Glasgow, UK

## Background

Asthma is the commonest long-term condition in children, affecting an estimated five million schoolchildren in Europe.^1^ The condition is responsible for substantial morbidity with 11% of children in the United Kingdom describing recurrent episodes of wheeze,^2^ and resulting in days lost from school and time lost from work for their parents/carers.^3^ Although in the United Kingdom, most school-age children with asthma are managed in primary care, each year there are in excess of 25,000 hospital admissions for children under the age of 14 years.^4^ Of the 195 asthma deaths investigated by the National Review of Asthma Deaths, 10 were in children under the age of 10 years.^4^

The American Thoracic Society/European Respiratory Society Task Force defines exacerbations (or asthma ‘attacks’) as events characterised by a change from the patient’s previous status, which may be severe (necessitating urgent action such as a course of steroids and/or hospitalisation) or moderate (prompting a temporary change of treatment).^5^ Many definitions include a fall in peak flow (e.g., to 50% or 75% of the patient’s best) though this may not be helpful in younger children or those unfamiliar with undertaking the manoeuvre,^6^ and may be indicative of poor asthma control rather than exacerbation.^7^ Frequent exacerbations in children not only have an impact on quality of life and school attendance but are also associated with an accelerated loss of lung function.^8,9^

Guidelines highlight that monitoring should not only include objective assessment of symptomatic asthma control with validated questionnaires (e.g., Asthma Control Questionnaire^10,11^ or Asthma Control Test^12,13^) or morbidity scores (e.g., Royal College of Physicians three questions^14^) but also include an assessment of the future risk of an exacerbation. Although recent work has devised a risk score for use in adults (16–60 years), with an area under the receiver operating characteristic curve for prednisolone use of 0.83,^15^ no such score exists for children aged 5–12 years.

A recent previous history of exacerbations, in particular, severe exacerbations,^16–21^ and persistent poor control^20,21^ are strongly associated with an increased risk of severe exacerbations, but many other predictors have been described in children and young people. Allergic sensitisation,^16,18,22–24^ seasonal variability,^17,19^ exposure to environmental tobacco smoke,^18,25^ no inhaled steroids^17,20,21^ regimes with multiple inhalers,^26^ impaired lung function^16,20^ and poor adherence to preventer medication^27^ have all been associated with increased risk of exacerbations. Other factors described in adults and teenagers (such as socioeconomic status,^28^ ethnicity,^29^ upper airways disease,^30^ blood eosinophilia,^31^ obesity^32,33^ and non-attendance for review appointments^4^) may also be relevant as predictors of exacerbations in young children. There is thus a need to identify and weight risk factors in children aged 5–12 years from factors identified in a systematic review, which can be further tested and validated in this population group.

The focus of this systematic review will be factors that increase a child’s propensity to asthma exacerbations, rather than immediate triggers for exacerbations, e.g., respiratory tract infections, exposure to airborne pollutants or allergens, physical or emotional exertion.

## Aims

To undertake a systematic review of the literature to:

1. Identify factors associated with the risk of asthma exacerbations in children aged 5–12 years.
2. Quantify their importance to inform the assessment of risk in children.

## Methods

We will follow the systematic review procedures described in the *Cochrane Handbook for Systematic Reviews of Interventions*.^34^

### ‘PICOS’ criteria

The PICOS criteria and study designs of interest are given in Table 1. We will include both controlled trials of interventions that aim to reduce exacerbation risk and observational studies, which seek to identify risk factors. We are interested in factors that contribute to future risk as opposed to immediate triggers (such as contracting an upper respiratory tract infection, or events such as thunderstorms). We will not include trials of pharmacological efficacy, or studies of unusual events or factors that cannot be routinely measured/tested or those that are not routinely available. Studies investigating risk factors for incidence/prevalence of asthma, asthma symptoms or objective measures of asthma activity (lung function, symptom scores, medication usage, health care utilisation) will not be included.

### Outcome measures

Our primary outcome will be severe exacerbations of asthma: asthma symptoms and/or objective evidence of airways obstruction outside the patients’ normal variation necessitating (a) a short course (at least 3 days) of oral corticosteroids and/or (b) a hospitalisation or emergency department visit requiring systemic corticosteroids.^5^

In addition, we are interested in risk factors for, and predictors of moderate exacerbations.^5^ See Table 1 for definitions.

### Identification of studies

The following electronic databases will be searched: MEDLINE, EMBASE, CINAHL, AMED, PsycINFO and CENTRAL. Unpublished and in-progress studies will be identified by the following: (a) searching internet-based trial registries, i.e., ClinicalTrials.gov (http://www.clinicaltrials.gov) and Current Controlled Trials (www.controlled-trials.com), and (b) contacting experts in the field. See Appendix 1 for an example of the search strategy developed for the MEDLINE database that will be adapted to search other databases. Papers included in related systematic reviews in adults will be checked to identify reports on children,^35^ and forward and backward citation checks will be undertaken on the included papers. In addition, our expert advisors will be asked to identify known and anticipated risk factors for asthma exacerbations in children as a reality check to ensure we have captured all likely predictors.

No language or publication time restrictions will be imposed. Translations will be undertaken where necessary, and papers for which translation is not feasible will be noted.

### Study selection and data extraction

Independently, two reviewers (NT or Audrey Buelo (AB) and SMcL) will perform the following:

- Review titles and abstracts of papers identified from the literature searches and select potentially relevant studies.
- Assess retrieved full texts of all potentially eligible studies against the review inclusion/exclusion criteria.
- Extract data using a customised data extraction form, piloted on a subsample to ensure the form is easily and consistently interpreted and captures all relevant information (including PICOS criteria, definitions used and outcomes).

Disagreements at each stage of the review will be resolved by discussion between the reviewers (NT/AB and SMcL) or, if necessary, arbitration by a third reviewer (HP). Unresolved issues relating to interpretation of inclusion/exclusion criteria will be referred to the Steering Group.

We will attempt to contact authors of the papers with missing or unclear essential information. Multiple publications from the same study will be treated as a single study, but draw on all the relevant publications.

The selection process will be summarised using a PRISMA flow diagram.^36^

### Assessment of the methodological quality

Two reviewers (AB and SMcL) will independently assess the methodological quality of the included papers. Using the Cochrane Risk of Bias Tool,^34^ randomised trials will be assessed for bias. Each individual domain (selection, performance, detection, attrition, reporting and other bias) will independently be judged by two reviewers (AB and SMcL) to be at low, unclear or high risk of bias. A summary assessment will include the overall risk of bias. Non-randomised studies will be assessed using the Newcastle-Ottawa checklist,^37^ which covers selection, comparability and outcome domains and is customised for cross-sectional, case–control and cohort studies. Authors will be contacted for unpublished information.^38^

### Data analysis and synthesis

We will provide a descriptive summary of the factors associated with a significant risk of exacerbation of asthma in children aged 5–12 years in detailed tables, and undertake a narrative synthesis of the data. We anticipate substantial heterogeneity of the studies and so do not plan to undertake a formal quantitative meta-analysis, but will weight identified factors as conferring slightly, moderately or greatly increased risk, based on the observed effect size and confidence intervals and the quality of the included papers, which will be interpreted in the light of biological plausibility. The weighting will be done independently by two raters. Disagreements will be resolved by discussion with a third rater arbitrating if necessary

The results across studies will be explored graphically and through summary measures to investigate whether the heterogeneity of effect can be accounted for by known factors. Severity of asthma and co-morbid rhinitis are part of the causal pathway for the outcome and are likely to be predictors in the final model and so will be investigated in an exploratory way.

The protocol is registered with PROSPERO International Prospective Register of Systematic Reviews (CRD42016037464), and the findings will be summarised and published in a peer-reviewed journal.

## Discussion

The key aim of the management of asthma in children (and all ages) is to reduce the burden of disease both by achieving good control of day-to-day symptoms and by reducing the risk of troublesome, and potentially serious asthma attacks. Targeting those at risk, has the potential to facilitate care commensurate with need and improve outcomes for those at highest risk. Likely risk factors include markers of severe disease and historical poor control (including previous exacerbations), as well as allergic sensitisation, exposure to environmental factors (including parental smoking), especially, the combination of infection and allergen exposure in sensitised children,^24^ and poor adherence to preventer medication and non-attendance for review appointments. Identification and weighting of these features of children and their family/environment will allow clinicians to identify ‘at-risk’ children, and inform discussions with parents about the need for regular ‘preventer’ treatment.

The vision is that risk assessment will have application both clinically for assessing individual children’s asthma control and thus focussing additional care on those at high risk, as well as for assessing control within populations and targeting care at high-risk populations.

## Figures and Tables

**Table 1:** PICOS criteria for the search strategy

Population	Children aged 5–12 years with doctor-diagnosed ‘active’ asthma (that is who have had a prescription for asthma treatment within the previous year), across all severities and degrees of control. We will include studies with a wider range of ages if results for children aged 5–12 years are reported separately or if >50% of the children are within this age range.

Intervention (if applicable)	Any intervention that aims to reduce exacerbation risk, specifically excluding trials of pharmacological efficacy as robust reviews are in existence for these. Examples might include interventions to improve medication-related behaviour (adherence, inhaler technique), social or lifestyle adaptation, improve residential environment (reduce housing damp/mould, improve indoor air quality) and reduce stress in mothers and children). Observational studies (cohort, case–control and cross-sectional) without a specific intervention that seek to identify relevant risk factors will also be included.

Control/comparator (if applicable)	Usual care.

Outcomes	Our primary outcome is severe exacerbations of asthma defined according to the ATS/ERS Task Force: asthma symptoms and/or objective evidence of obstruction outside the normal variation for the patient necessitating (a) a short course (at least 3 days) of oral corticosteroids and/or (b) a hospitalisation or emergency department visit requiring systemic corticosteroids.^5^
	Moderate exacerbations as defined by the ATS/ERS task force (asthma symptoms and/or airflow obstruction) outside the normal variation for the patient prompting a temporary change of treatment (excluding systemic steroids) to prevent a severe exacerbation.^5^

Setting	Any setting

Study designs	Randomised controlled trials, controlled clinical trials, interrupted time series, controlled before-and-after studies, cohort and case-controlled studies (but not case studies or case series).

Abbreviation: ATS/ERS, American Thoracic Society/European Respiratory Society.

**Table A1:** Ovid MEDLINE(R) 1946 to present with daily update—executed 10 May 2016

*#*	*Search statement*	*Results*
1	exp Asthma/ or Bronchial Spasm/ or exp Bronchoconstriction/ or (asthma* or wheez* or bronchoconstrict* or bronchial constrict* or bronch* constrict* or bronchial spasm or bronchospas*or bronch* spasm*).tw.	150958
2	Bronchial Hyperreactivity/ or Respiratory Hypersensitivity/	15662
3	1 or 2	157160
4	(exacerb* or deteriorat* or aggravate* or acute* or status* or sever* or wors* or attack* or crisis or critical or hospital* or relapse or uncontrolled or poor* controlled).tw. or exp Recurrence/ or exp Disease Progression/ or exp Mortality/ or exp Death/	4817367
5	exp Emergency Service, Hospital/ or exp Emergency Medical Services/ or exp Hospitalisation/ or exp Hospitals/ or exp Intensive Care Units/ or exp Emergencies/ or (emergenc* or acute care or intensive care or intensive treatment unit* or hospital*).tw.	1291812
6	(admission* or admit* or attend* or visit* or present* or utilis* or utiliz* or use* or using).tw.	8649008
7	5 and 6	708396
8	exp Primary Health Care/ or exp General Practice/ or exp Family Practice/ or exp Physicians/ or (physician* or doctor* or health care professional or general practice or asthma nurse or specialist nurse or GP).tw.	574496
9	exp ‘Appointments and Schedules’/ or appointment*.tw. or visit*.tw.	165155
10	(unscheduled or additional or increase*).tw.	3829910
11	8 and 9 and 10	9990
12	exp Medicine/ or exp Therapeutics/ or exp Anti-Asthmatic Agents/ or exp Bronchodilator Agents/ or exp Adrenergic beta-Agonists/ or exp Cholinergic Antagonists/ or exp Steroids/ or exp Glucocorticoids/ or (medicin* or treatment or medication or steroid* or corticosteroid* or glucocorticosteroid* or inhaler* or beta agonist* or beta-2-agonist* or SABA or anticholinergic* or ICS).tw.	7354258
13	(rescue or supplement* or (step* adj up) or adjuvant or additional or increase*or augment*).tw.	849246
14	12 and 13	379948
15	exp Steroids/ or exp Glucocorticoids/ or (steroid* or corticosteroid* or glucocorticosteroid*).tw.	923905
16	(systemic or oral or intravenous or intramuscular or injectable or parenteral or IV or IM).tw. or exp Injections, Intravenous/ or exp Administration, Intravenous/ or exp Injections, Intramuscular/ or exp Infusions, Parenteral/ or rescue.tw.	1384151
17	15 and 16	120669
18	(((nocturnal or night* or sleep*) adj2 (symptom* or wheeze* or asthma or wakening or woken or disturb*)) or ((daily or daytime) adj2 (activit* adj2 disturb*))).tw.	15029
19	exp Oxygen Inhalation Therapy/ or exp Intubation, Intratracheal/ or nebuli*.tw.	63956
20	((increas* or wors* or aggravate*) and (frequenc* or sever* or symptom* or wheez* or breathless* or dyspn?a)).tw.	870900
21	4 or 7 or 11 or 14 or 17 or 18 or 19 or 20	5466020
22	3 and 21	67554
23	Risk Factors/ or Risk/ or (risk adj3 factor*).tw. or predict*.tw. or (risk* adj2 exacerb*).tw.	1719001
24	23 and 22	12770
25	Animals/ not Humans/	4196538
26	24 not 25	12662
27	Adult/ not Child/	3672244
28	26 not 27	8379
29	(COPD.tw. or Pulmonary Disease, Chronic Obstructive/) not Asthma/	31916
30	28 not 29	8099
31	Comment/ or Letter/ or Editorial/ or Autobiography/ or Biography/ or Bibliography/ or Dictionary/ or Directory/ or Interactive Tutorial/ or Lectures/	1578832
32	30 not 31	7989

## Appendix 1

Table A1
